# *Clostridium difficile* and methicillin-resistant *Staphylococcus aureus* shedding by slaughter-age pigs

**DOI:** 10.1186/1746-6148-7-41

**Published:** 2011-07-26

**Authors:** J Scott Weese, Joyce Rousseau, Anne Deckert, Sheryl Gow, Richard J Reid-Smith

**Affiliations:** 1 Dept of Pathobiology, University of Guelph, Guelph, Ontario, Canada; 2 Laboratory for Foodborne Zoonoses, Public Health Agency of Canada, Guelph, Ontario, Canada; 3 Dept of Large Animal Clinical Sciences, University of Saskatoon, Saskatoon, Saskatchewan, Canada; 4 Laboratory for Foodborne Zoonoses, Public Health Agency of Canada, Saskatoon, Saskatchewan, Canada

## Abstract

**Background:**

*Clostridium difficile *and methicillin-resistant *Staphylococcus aureus *are critical human pathogens and of increasing concern in food animals. Because of the apparent impact of age on prevalence of these organisms, studies of slaughter age pigs are important when considering the potential for contamination of food. This study evaluated *C. difficile *and MRSA shedding by slaughter age pigs from farms across Canada.

**Results:**

*Clostridium difficile *was isolated from 30/436 (6.9%) samples from 15/45 (33%) farms. After adjusting for clustering at the herd level, the prevalence was 3.4%. Ribotype 078 (toxinotype V, North American Pulsotype 7) was the most common strain, accounting for 67% of isolates. MRSA was isolated from 21/460 (4.6%) pigs from 5/46 (11%) farms. The prevalence in pigs after adjusting for clustering at the herd level was 0.2%. Seven different spa types were identified, with 3 related spa types (t011, t034, new) accounting for 16 (76%) consistent with ST398 predominating.

Both MRSA and *C. difficile *samples were collected from 45 farms. Both MRSA and *C. difficile *were detected on 2 (4.4%), with *C. difficile *only on 13 (29%), MRSA only on 3 (6.7%) and neither on 27 (60%).

**Conclusions:**

The prevalence of *C. difficile *and MRSA in slaughter age pigs was relatively low, particularly in comparison with studies involving younger pigs. The predominance of *C. difficile *ribotype 078 and MRSA ST398 was not surprising, but there was diversity in strain types and the majority of isolates of both organisms were strains that can be found in humans. While the prevalence of *C. difficile *and MRSA in slaughter age pigs was relatively low, there is clearly potential for contamination of meat from healthy pigs carrying this pathogen into slaughterhouses.

## Background

*Clostridium difficile *and methicillin-resistant *Staphylococcus aureus *are important causes of disease in humans and of increasing concern in food animals. In pigs, *C. difficile *infection (CDI) can cause severe enteritis in young (1-7d old) piglets, often with high mortality [1-3]. Human CDI appears to be increasing in incidence and severity internationally [4-6]. Additionally, while once considered mainly a hospital-associated pathogen, community-associated CDI (CA-CDI) appears to be increasing [7,8] and toxinotype V strains, particularly ribotype 078, appear to be over-represented in CA-CDI [9,10]. These strains have predominated in studies of pigs and cattle [11-16] and have been found in retail meat [17,18], raising concerns that *C. difficile *might be a zoonotic and foodborne infection [13,19,20]. Highly variable (0-52%) shedding rates have been reported in studies of healthy pigs [12,15,16,21,22]. However, studies reporting high prevalences have involved young piglets and there is evidence of a significant impact of age on *C. difficile *shedding [15,23]. Evaluation of food contamination risks requires an understanding of the prevalence of *C. difficile *shedding and the *C. difficile *types shed by slaughter-age pigs, not piglets.

Similarly, methicillin-resistant *Staphylococcus aureus *(MRSA) was once predominantly a hospital-associated pathogen in humans, but has emerged as an important community-associated pathogen internationally. Recently, livestock-associated MRSA (LA-MRSA), caused by sequence type 398 (ST398) strains [24-26], has emerged as an important public health issue, particularly in Europe. MRSA colonization has been identified in healthy pigs from various countries, sometimes at high rates [25-30]. Similarly, high MRSA colonization rates have been reported in pig farmers [29,31,32], and associations between pig contact and both MRSA infection and MRSA colonization in humans have been reported [33-38]. MRSA has also been identified in retail meat [39-41], heightening concerns but currently with unclear public health significance.

Prevalence studies have been reported for different pig populations in different regions, with prevalence ranging from 1 to 80% [25,28,30,42,43]. As with *C. difficile*, there is evidence of age-related changes in MRSA colonization rates [44] and study of pigs at the age of slaughter is most relevant for assessment of food contamination issues. Studies involving commingled pigs, pigs at slaughterhouses or from multiple farms from the same production systems have been performed [28,30], but could introduce effects of clustering or transient contamination from transportation and may therefore not provide an optimal estimate of true population prevalence. For these reasons, studies of non-commingled pigs close to the age of slaughter from a large number of unassociated farms are required to obtain a better estimate of the prevalence of MRSA colonization in pigs that are ready to enter the food chain.

The objectives of this study were to determine the prevalence of *C. difficile *and MRSA shedding by slaughter-age pigs on farms across Canada and to characterize recovered isolates.

## Methods

### Study Population and Sample Collection

Commercial swine farms from across Canada were recruited in conjunction with the Canadian Integrated Program for Antimicrobial Resistance Surveillance (CIPARS). This program has a network of farms and veterinarians across Canada that participate in ongoing surveillance and periodic research studies. Veterinarians participating in CIPARS were contacted and asked to recruit herds. On each participating farm, freshly passed fecal samples were collected from pens containing grower-finisher pigs close to the time of slaughter. A single sample was collected per pen, to represent an individual pig sample. The target was 10 pens per farm, however on some farms, 10 separate pens were not available so a smaller number of samples was obtained. Additionally, nasal swabs were collected from 10 non-comingled grower-finisher pigs that were close to slaughter age. Fecal and nasal samples were not necessarily collected from the same pigs, so *C. difficile *and MRSA were analysed independently.

### *Clostridium difficile *isolation

Approximately 1 g of feces was inoculated into 9 ml of *C. difficile *agar base (Oxoid Company, Nepean, Canada) with *C. difficile *moxalactam norfloxacin supplement (Oxoid Company, Nepean, Canada)(CDMN) and 0.1% sodium taurocholate and incubated anaerobically at 37°C for 7 days. An aliquot of the broth was alcohol shocked with an equal volume of anhydrous ethanol for 1 hour. This mixture was then centrifuged for 10 min at 4000 rpm. The supernatant was discarded and the pellet was streaked onto a CDMN agar plate and incubated anaerobically at 37°C for 48 h. Suspicious colonies were subcultured onto blood agar and confirmed as *C. difficile *by Gram stain appearance, colony morphology, characteristic odor and production of l-proline aminopeptidase.

### *Clostridium difficile *isolate characterization

Isolates were typed by PCR ribotyping as has been described elsewhere [45]. In situations where the ribotype was known to be a recognized international ribotype through previous typing of reference strains from the PHLS Anaerobic Reference Unit (Cardiff, UK), the appropriate numerical designation (i.e. 078) was used. Otherwise, internal nomenclature was used. Genes encoding production of toxins A (*tcdA*) and B (*tcdB*) were evaluated using PCR [46,47]. Detection of CDT (binary toxin) was performed using PCR directed at *cdtB*, the binding component [48]. Toxinotyping [49] and pulsed field gel electrophoresis (PFGE) [50] were performed on a representative of each toxigenic ribotype. Sequence analysis of *tcdC *was performed and interpreted as previously described [51].

### MRSA Isolation

Nasal swabs were inoculated into 9 ml of enrichment broth consisting of 10 g tryptone/L, 75 g sodium chloride/L, 10 g mannitol/L and 2.5 g of yeast extract/L. After 24 h incubation at 35°C, 50 ul of broth was inoculated onto MRSA Chromogenic agar (BBL CHROMagar MRSA, Becton, Dickinson and Company, Sparks, MD). Plates were incubated at 35°C and read after 24 and 48 h. Isolates were identified as *S. aureus *by colony morphology, Gram stain appearance, catalase reaction, coagulase reaction and *S. aureus *latex agglutination test (Pastorex Staph-plus, Bio-Rad, France). Methicillin-resistance was confirmed by penicillin binding protein 2a latex agglutination test (MRSA latex agglutination test, Oxoid Ltd., Hants, UK).

### MRSA Isolate Characterization

Isolates were typed by sequencing of the x region of the protein A gene (spa typing) [52] and classified using the Ridom system (http://www.spaserver.ridom.de). Real time PCR was used to detect the *lukF *and *lukS *components of the Panton-Valentine leukocidin (PVL) [53]. Positive and negative controls were performed with each PCR run.

### Statistical Analysis

The crude prevalence was calculated for both *C. difficile *and MRSA. The prevalence was then adjusted for clustering at the herd level using Generalized Linear and Latent Mixed Models (GLAMM) with adaptive quadrature (Stata Intercooled version 10.1, Stata Corporation, College Station, Texas, USA). Chi-squared test was used to compare the herd level prevalence between provinces. A *P *value of < 0.05 was considered significant.

This study was approved by the University of Guelph Animal Care Committee.

## Results

### Clostridium difficile

*Clostridium difficile *was isolated from 30/436 (6.9%) samples from 15/45 (33%) farms (Table 1). The prevalence after adjustment for clustering at the herd level was 3.4%. Five to 10 samples were collected per farm (median 10). There was a significant difference between provinces at the farm level (*P *= 0.002), with the farm prevalence ranging from 0-100%. On positive farms, between 1 and 5 samples (median = 1), representing between 10-100% of samples, were positive.

**Table 1:** Prevalence of *Clostridium difficile *shedding by slaughter age pigs in 5 difference Canadian provinces.

Province	Number of pigs	Unadjusted prevalence (%) (95% CI)	Adjusted pig prevalence (%), (95% CI)	Farm prevalence
A	100	4.0 (1.5-10.2)	3.0 (0.5-15.9)	3/11 (27%)

B	50	26 (15.7-39.8)	26 (15.7-39.8)	5/5 (100%)

C	100	4.0 (1.5-10.2)	4.0 (1.5-10.2)	4/10 (40%)

D	106	0	0	0/11

E	80	11.3 (6.0-20.2)	3.0 (0.2-31.4)	3/8 (38%)

Total	436	6.9 (4.9-9.7)	3.4 (0.8-13.6)	15/45 (33%)

Seven different ribotypes were identified (Table 2). Ribotype 078 isolates were classified as North American Pulsotype (NAP) 7 by PFGE. One additional toxinotype V ribotype was indistinguishable from NAP7 on PFGE, however the other toxinotype V ribotype (S6) had a PFGE pattern that is not consistent with any NAP type and had a 5 band difference from NAP7. Three of the 6 toxigenic ribotypes 3 (50%), accounting for 82% of isolates, have been previously identified in humans in Canada [54].

**Table 2:** *Clostridium difficile *isolated from healthy slaughter age pigs in Canada.

Ribotype	n (%)	TT	PFGE	Toxin genes	*tcdC*	Farms	Provinces
078	20 (67%)	V	NAP7	*tcdA, tcdB, cdtB*	39 bp deletion, C184T mutation	12	A, B, C, E

S6	3 (10%)	V	NAP7-like	*tcdA, tcdB, cdtB*	39 bp deletion, C184T mutation	2	E

MOH-S	2 (6.7%)	0	Non-epidemic clone	*tcdA, tcdB*	Wild-type	1	A

R	1 (3.3%)	0	Non-epidemic clone	*tcdA, tcdB*	Wild-type	1	C

S7	1 (3.3%)	V	NAP7	*tcdA, tcdB, cdtB*	39 bp deletion, C184T mutation	1	E

OVCAA	1 (3.3%)	0	Non-epidemic clone	*tcdA, tcdB*	18 bp deletion	1	A

OVCJ	2 (6.7%)	NA	NT	None	NA	2	B

TT: Toxinotype

NA: not applicable

NT: Not tested

On 7/15 (47%) positive farms, *C. difficile *was recovered from more than one pig. On 3 of these, all isolates were the same; ribotype 078. On 2 farms, there was a combination of ribotype 078 and a single nontoxigenic isolate. On 2 other farms, ribotype 078 plus 1 or 2 other toxinotype V ribotypes were found. No disease attributed to *C. difficile *was reported on any of the participating farms.

### MRSA

MRSA was isolated from 21/460 (4.6%) pigs from 5/46 (11%) farms (Table 3). The prevalence in pigs after adjusting for clustering at the herd level was 0.2%. The on-farm prevalence ranged from 0-70%, with a range of 20-70% for positive farms. There was not a statistically significant difference in farm prevalence between provinces (*P *= 0.35)

**Table 3:** Isolation of methicillin-resistant *Staphylococcus aureus *(MRSA) from slaughter-age pigs in Canada.

Province	Number of farms, pigs	Unadjusted pig prevalence (%, 95% CI)	Adjusted pig prevalence (%, 95% CI)	Farm prevalence
A	10, 100	0	0	0

B	5, 50	0	0	0

C	10,100	11 (6.2-18.8)	0.04 (0.0-99.8)	30% (3/10)

D	13, 130	0.8 (0.1-5.3)	0.77 (0.1-5.3)	7.7% (1/13)

E	8, 80	11.3 (6.0-20.2)	0.35 (0-88.8)	25% (2/8)

Total	46, 460	4.6 (3.0-6.9)	0.002 (0-12.8)	13% (6/46)

Seven different spa types were identified, corresponding to three different clones (Table 4). All isolates were PVL negative. Three related spa types (t011, t034, 04652), accounting for 16 (76%) isolates were consistent with ST398. Three spa types, t002 (n = 2), t5518 (n = 1) and t067 (n = 1) were consistent with Canadian epidemic MRSA-2 (CMRSA-2). One additional spa type (t064) was unrelated to the others and was consistent with CMRSA-5.

**Table 4:** Typing data for methicillin-resistant *Staphylococcus aureus *(MRSA) isolated from slaughter-age pigs in Canada.

Spa type	n	Repeat pattern	Farms	Province
t011	8 (38%)	08-16-02-25--------------34-24-25	5, 9	C

t034	7 (33%)	08-16-02-25-02-25-----34-24-25	28	E

t4652	1 (4.8%)	08-16-02-25-02-25-------- 24-25	9	C

				

t002	2 (9.5%)	26-23-17-34-17------20-17-12-17-16	13, 24	D, E

t5518	1 (4.8%)	26-23-17-34-17-23------17-12-17-16	5	E

t067	1 (4.8%)	26-23-17-34-17------20-17-12-17	5	E

				

t064	1 (4.8%)	11-19-12-05-17-34-24-34-22-25	13	E

A single spa type was found on two farms, one with 7 t034 isolates and one with a single t002 isolate. On the 3 other farms, two or 3 different spa types were identified.

### Combined

Both MRSA and *C. difficile *samples were collected from 45 farms. Both MRSA and *C. difficile *were detected on 2 (4.4%), with *C. difficile *only on 13 (29%), MRSA only on 3 (6.7%) and neither on 27 (60%).

## Discussion

This study has identified a relatively low prevalence of both *C. difficile *and MRSA in pigs shortly before the time of slaughter. These data are consistent with recent studies demonstrating a significant impact of age on *C. difficile *colonization in pigs [15,22,23,55], such a a longitudinal study that reported a 96% cumulative prevalence in young piglets, with colonization of 74% of piglets on day 2 of life but only 3.7% in the same piglets on day 62 [23], and a similar study in an integrated swine operation reported *C. difficile *shedding in 50% of suckling piglets, but only 3.9% of grower-finisher pigs and breeding animals [15]. The reason for variation in prevalence with age has not been specifically evaluated but may relate to the poorly developed intestinal microflora in young pigs, with more competition from other microorganisms as the pig ages.

The relatively low farm- and pig-prevalence of MRSA was somewhat surprising, although some of the adjusted prevalence estimates had wide confidence intervals. These data are in contrast to a previous Canadian study that reported MRSA from 25% of pigs (piglets, weaners, grower-finishers) and 45% of farms in Ontario, Canada [29], as well as some studies from other regions reporting colonization rates of 39-80% [25,28,30]. However, results are similar to the report of isolation of MRSA from 1.3% of pigs in Switzerland at the time of slaughter [42], as well as 0.8% in weaned pigs in Malaysia [56]. As with *C. difficile*, there appears to be a significant impact of age on MRSA colonization in pigs, with MRSA colonization rates decreasing dramatically as pigs age [44], and this may account for the low prevalence reported here, perhaps because of changes in the nasal commensal microflora or immune response.

The inter-provincial difference in prevalence in *C. difficile *was unexpected. There were no readily apparent explanations, however specific farm management factors were not queried because of the relatively small farm sample size. Identification of factors that may influence differences in *C. difficile *between farms or between regions is important, in order to identify potential interventions for the reduction of *C. difficile *shedding.

Regardless of the reasons, the relatively low prevalence of *C. difficile *and MRSA in pigs approaching the time of slaughter may have implications for assessment of foodborne contamination. While the prevalence in young piglets may have pig-health implications (at least for *C. difficile*), the relevance for foodborne contamination is limited because young piglets are rarely slaughtered for food. The prevalence of *C. difficile *or MRSA in pigs at or shortly before the time of slaughter is presumably more relevant, and this study indicates a relatively low prevalence. Additionally, the potential for marked variation in prevalence simply as a factor of age must be considered in studies involving *C. difficile *and MRSA in pigs and likely other animal species.

Despite the relatively low prevalence, the potential for food as a source of *C. difficile *and MRSA for humans cannot be dismissed. Contamination of retail meat clearly can occur [17,39-41,57-59], but limited information is available regarding the origins of food contamination. Prospective studies of the slaughter and processing systems are required to determine the source, or sources, of contamination and identify potential interventions.

*Clostridium difficile *typing data were not particularly surprising. Ribotype 078, a toxinotype V strain, predominated, as has been previously reported in studies of pigs [12-14,21], but there were two other toxinotype V ribotypes. Non-ribotype 078 toxinotype V strains have been reported previously in pigs, particularly ribotype 066 [12,16,21]. Ribotype 066 was not identified in this study and the lack of a standard international system for identifying and naming uncommon ribotypes hampers comparison of uncommon isolates from different studies. It is, therefore, unclear whether the two non-ribotype 078/066 ribotypes identified here have been reported in pigs elsewhere. It is likely that these two strains are closely related to, and probably evolved from, ribotype 078. Alterations in *tcdC*, a gene that down regulates production of toxins A and B has been linked to hypervirulence in some *C. difficile *strains, particularly ribotypes 027 and 078 [10,60], although the true role of this gene in virulence is still unclear. Ribotype 078, S6 and S7 possessed the expected 39 bp deletion and C184T nonsense mutation, providing further support to the suspicion that they are closely related.

Similarly, the predominance of spa types that correspond to ST398 was not unexpected, given the predominance of this clone in pigs in most regions [25,28,30,61]. However, while ST398 has accounted for virtually all isolates reported in pigs from many regions, other strains have been previously identified in pigs [27,29]. In this study, two human epidemic clones were identified. The first, CMRSA-2 (also known as USA100) is an ST5 strain that is the most common cause of hospital-associated MRSA infection in humans in Canada [62], as well as the most common strain found in colonized humans in the US [63]. It accounted for 14% of isolates in an earlier study of pigs in Ontario [29] and 15% in this study, but has not, to our knowledge, been reported in pigs elsewhere. Interestingly, this strain accounted for 29% and 100% of MRSA isolates recovered from pork in two recent studies of retail meat in Canada [40,41]. The other strain was spa type t064 that corresponds to CMRSA-5 (USA500), an ST8 strain that is a relatively uncommon human epidemic clone [63], but commonly reported in horses in some regions [29]. This strain has not, to our knowledge, been previously isolated from pigs, however it accounted for 38% of MRSA isolates in a study of Canadian retail pork [41]. The presence of both livestock-associated and human epidemic clones suggests that there may be multiple routes of MRSA exposure in pigs, both from other pigs and from humans. ST398 MRSA can reasonably be assumed to have originated in livestock, although humans can carry this strain and could presumably pass it between pigs. The human epidemic clones found in pigs almost certainly were ultimately from humans, since parallel development of these strains in pigs is extremely unlikely. However, it is clear that both livestock associated and human MRSA clones can be transmitted within and between pigs and humans.

No pigs enrolled in this study had signs of clinical MRSA infection. While MRSA can cause disease in pigs [64], clinical infections appear to be quite rare, something that is positive from a pig health standpoint but which allows MRSA to be present undetected on farms.

Interestingly, the presence of both MRSA and *C. difficile *on a farm was uncommon, being identified in only 4.4% of farms. The reasons for this are unclear but since MRSA and *C. difficile *share various risk factors in humans (e.g. antimicrobial administration), it is somewhat surprising that there was no apparent association between the presence or absence of these two pathogens on farms.

## Conclusions

Both *C. difficile *and MRSA were identified in a small percentage of pigs at the age of slaughter, consisting mainly of strains that are of concern for human health. The public health risk posed by *C. difficile *and MRSA in Canadian pigs is unclear, partly because of poor understanding of the role of livestock associated MRSA and *C. difficile *strains in community-associated disease in people in the country. ST398 MRSA infections in humans appear to be rare in Canada, although cases have been recently identified and there is concern that they could increase [65]. The presence of these two pathogens in pigs, albeit at relatively low prevalence, does not necessarily indicate a human health risk, either from direct contact or foodborne contamination, however it should not be dismissed. A better understanding of the epidemiology of *C. difficile *and MRSA in livestock and humans is required to help elucidate the role of animals in human infections and to identify possible interventions to reduce the potential public health impact of this important pathogen.

## Authors' contributions

JSW and RRS conceived the study, and JSW, AD, SG and RRS participated in study design. AD and SG coordinated field sampling and communication with participating veterinarians. JR performed all laboratory studies. JSW, AD and SG analysed the data. JSW wrote the initial draft of the manuscript. All authors participated in manuscript review. All authors read and approved the final manuscript.
